# On a Case of Fistula of the Urethra

**Published:** 1851-09

**Authors:** John Patteson


*On a case of Fistula of the Urethra.* By JOHN PATTESON, Esq., M. D.—M. I——, aged twenty-four, consulted me on Jan. 3, 1851, complaining of a muco-purulent discharge from the urethra. He had contracted gonorrhœa nine months previously, and the discharge since then has been constant. On examining the urethra with a bougie, I found a stricture in the membranous portion, and a sac or tumor in the portion about an inch below the corona glandis. This sac or tumor, when he made water, became always more or less distended, and by pressure could be nearly emptied; a sanious muco-purulent discharge appearing at the urethral orifice.

I punctured the cyst, and threw an injection of tincture of iodine diluted with water into it daily, at the same time treating the stricture in the usual manner. To the patient, who was of the scrofulous diathesis, I gave full doses of the iodide of potassium, and the fluid extract of sarsaparilla. This treatment along with bandaging, was persevered in for some time, without any benefit to the urethral fistula. On Feb. 22, I operated for the cure of his fistulous opening in the following manner, being assisted by my pupil, Mr. Constantine M'Guire:

Having first introduced a full-sized silver sound into the urethra, I then, with a narrow sharp-pointed knife, laid open the sac, and then found three fistulous canals proceeding from the cyst, in a direction downwards towards the perinæum, to the extent of three-quarters of an inch, when all three united, becoming one canal, which took a direction immediately backwards, and opened into the urethra, by a single opening rather more than one line in diameter. The next step was carefully to excise my puncture of the sac, and the callous sides of the fistulous canals; I then, with a small cautery-iron, touched the opening in the urethra. For the first few days the cold-water dressing was used, and the bladder regularly emptied with the catheter. The after-dressing was the basilicon ointment, and now and then with a solution of nitrate of silver.

In the course of ten days all discharge from the urethra had ceased; the patient progressed favorably, and on March 16th he was discharged cured.—*London Lancet.*

